# Analysis of climate factors and dengue incidence in the metropolitan region of Rio de Janeiro, Brazil

**DOI:** 10.1371/journal.pone.0251403

**Published:** 2021-05-20

**Authors:** Leandro Layter Xavier, Nildimar Alves Honório, José Francisco Moreira Pessanha, Paulo César Peiter

**Affiliations:** 1 Parasitic Diseases Laboratory, Tropical Medicine Program, Oswaldo Cruz Institute, Oswaldo Cruz Foundation, Rio de Janeiro, Brazil; 2 Hematozoan Transmitting Mosquito, Tropical Medicine Program, Oswaldo Cruz Institute, Oswaldo Cruz Foundation, Rio de Janeiro, Brazil; 3 Institute of Mathematics and Statistics, State University of Rio de Janeiro, Rio de Janeiro, Brazil; Faculty of Science, Ain Shams University (ASU), EGYPT

## Abstract

Dengue is a re-emerging disease, currently considered the most important mosquito-borne arbovirus infection affecting humankind, taking into account both its morbidity and mortality. Brazil is considered an endemic country for dengue, such that more than 1,544,987 confirmed cases were notified in 2019, which means an incidence rate of 735 for every 100 thousand inhabitants. Climate is an important factor in the temporal and spatial distribution of vector-borne diseases, such as dengue. Thus, rainfall and temperature are considered macro-factors determinants for dengue, since they directly influence the population density of Aedes aegypti, which is subject to seasonal fluctuations, mainly due to these variables. This study examined the incidence of dengue fever related to the climate influence by using temperature and rainfall variables data obtained from remote sensing via artificial satellites in the metropolitan region of Rio de Janeiro, Brazil. The mathematical model that best fits the data is based on an auto-regressive moving average with exogenous inputs (ARMAX). It reproduced the values of incidence rates in the study period and managed to predict with good precision in a one-year horizon. The approach described in present work may be replicated in cities around the world by the public health managers, to build auxiliary operational tools for control and prevention tasks of dengue, as well of other arbovirus diseases.

## Introduction

Arthropod-borne diseases such as dengue, chikungunya, West Nile, Zika and yellow fever represent important emerging and re-emerging infectious diseases, which are considered serious global public health problems [1, 2]. Dengue is currently considered the most important mosquito-borne arbovirus infection to man, taking into account both its morbidity and mortality [3].

The World Health Organization (WHO) characterizes dengue as one of the world’s leading public health problems, with an estimated 50 to 100 million people infected annually, resulting in approximately 250,000 to 500,000 cases of haemorrhagic fever and 24,000 deaths / year in 100 countries (with the exception of Europe) [4–6]. In addition, total costs, totaling direct and indirect expenses resulting from this disease worldwide, reached 8.9 billion US dollars annually [7].

Dengue is an acute infectious disease caused by viruses (DENV) of the RNA genome, belonging to the Flaviviridae family and to the genus Flavivirus. To date, four antigenically distinct serotypes DENV-1, DENV-2, DENV-3, DENV-4 have been identified and a fifth serotype of dengue has been discovered in the State of Sarawak, located on the island of Borneo in Malaysia of wild transmission, DENV-5. This new serotype has been detected in humans and is associated with even severe cases [8, 9], but it has not yet been detected in Brazil.

The transmission of the dengue virus to man occurs through the bite of females of the genus *Aedes*, the main one being *Aedes (Stegomyia) aegypti* (Linnaeus, 1762), which presents urban and anthropophilic behavior, since it lives in cities and close to humans [10]. In addition to this species, in southeastern Asia, there is another primary vector of the dengue virus, *Aedes (Stegomyia) albopictus* (Skuse, 1894). In Brazil, *Ae*. *albopictus* is considered a potential vector of dengue viruses [11].

*Aedes aegypti* is associated to urban and suburban environments, where there is high human population concentration and high concentration of residences [12]. This species often shows an endophilic behavior, using the interior of the houses to be sheltered and, therefore, it is more often found in the intradomicile than in the peridomicile [12]. Their breeding grounds are usually artificial containers filled with rainwater or domestic water, including tires, cans, glasses, bottles, pots, water boxes, vats, brass, swimming pools, abandoned aquariums, among others [13–15].

The transmission of dengue is essentially urban. In this environment, all the key factors for its occurrence can coexist: man, the virus and the vector. In a situation where there is a simultaneous combination of socio-environmental and climatic conditions favorable to the mosquito’s reproduction, its population may explode [16].

Tropical countries are most affected because of their environmental, climatic and social conditions [17]. Climate is an important factor in the temporal and spatial distribution of vector-borne diseases such as dengue. Thus, rainfall and ambient temperature are called macro factors that are determinant for dengue, as they directly influence the population density of Ae. aegypti, which undergo seasonal fluctuations, due to these main variables. However, in areas of tropical or subtropical climate, its proliferation is continuous, although during the period of lower precipitation and lower average temperatures, the population density of this vector tends to reduce significantly [17, 18].

Studies of climatic variables can improve knowledge and prediction of epidemic seasonality because the vector-climate relationship is as important as vector-human interaction. Research in epidemiology has increasingly sought to find statistical and mathematical models based on climate factors that may explain the dynamics of dengue incidence. It is mainly tried to identify models with a strong future predictive power of dengue incidence to subsidize public health managers [3, 19]. In this sense, several authors study the relationship between climate variables and dengue, often using time-series analyzes to describe temporal trends, identify patterns and even make predictions [17, 20–24].

The present study has a nature similar to the works in the literature listed above, with the final objective of building a useful instrument for prediction of the disease and, in particular, of the occurrence of epidemics in the municipality of Duque de Caxias, in the Metropolitan Region of Rio de Janeiro, which borders the north of this city. The chosen climate data, namely temperature and rainfall, were obtained by remote sensing via artificial satellites. This type of data has important properties for epidemiological studies, especially in poor countries: they were previously available cost free for the whole world and they do not have deleterious missing values. To achieve this goal, we try to adjust an ARMAX model, the family of autoregressive models of moving averages (ARMA model) with exogenous variables, an approach for analysis and forecasting time series, which is an adequate way to treat the relationship between the incidence of dengue fever and climate variables, e.g., temperature and rainfall [25]. The ARMAX model is very flexible and can be extended to model non-stationary time series with seasonal components. In this case we have a SARIMAX model.

The most innovative point in the study was the utilization of climate data coming from remote sensing via artificial satellites, which offers better accuracy and consistency properties with a low cost. Due to the good quality of the adjustment and good predictive capacity of the model, the forecasts provided by the developed model offer valuable information so that public managers can improve public health surveillance systems and act preventively in order to mitigate or avoid future dengue epidemics [19]. However, the most critical limitation of the study was to use data series with a monthly periodicity, because thirty days is a very long time interval, in which the vector population can explode. Thus, it is advisable to use a shorter time interval, as a weekly or daily one, to allow more efficient and fast prevention actions by the public health managers. Moreover, the inclusion of other explanatory variables, such as wind, population of mosquitoes or people flow, can improve the performance of the ARMAX model.

## Materials and methods

### Study area

The present study analyses the relationship between dengue incidence and climate variables, such as temperature and rainfall, in the municipality of Duque de Caxias, State of Rio de Janeiro. This is an statistical model based on historical series of cases of dengue infection confirmed and reported in this municipality from January 2007 to December 2016.

The choice of this municipality is due to its location in the Baixada Fluminense, a region of high population density and marked by recurrent dengue epidemics. Because it is a metropolitan municipality bordering the capital of Rio de Janeiro, there is a strong daily flow of people that circulate between the two cities, being able to be carriers of the dengue virus. Add to this the economic importance of Duque de Caxias, the third largest GDP of the state [26].

This municipality is located in coordinates 22°C 47’ 08” S and 43° 18’ 42” W and has an area of 467.6 km^2^, being at a distance of 12 km from the city of Rio de Janeiro.

The population of Duque de Caxias in 2010 was 855,048 inhabitants, with 99.7% living in the urban area, while only 2,910 (a 0.32% proportion) live in rural areas. This municipality is located in the metropolitan region of Rio de Janeiro, which has a total population of over 12.5 million inhabitants [27].

### Data sources

Dengue is one of the notifiable diseases, according to the Brazilian Ministry of Health guidelines. Thus, any case of this disease in any public or private health unit must be notified [28].

The epidemiological data, the dengue cases reported in Duque de Caxias, comprising the period of 2007 to 2016, were obtained from the State Department of Health of the State of Rio de Janeiro (SES-RJ). Population data was obtained from IBGE (the Brazilian Institute of Geography and Statistics).

The climate data, namely temperature and rainfall, were obtained by the remotely sensed products here-in named as MODIS (Moderate Resolution Imaging Spectroradiometer), which is acquired from the satellites Aqua and Terra owned by NASA (North America Space Agency), and TRMM (Tropical Rainfall Measuring Mission), a collaboration of NASA with JAXA (Japan Aerospace Exploration Agency). Both products are available at NASA (https://mirador.gsfc.nasa.gov).

More specifically, the daily product MOD11A2 Version 6 at the 1-km spatial resolution has been used to address land surface temperature [29]. The spatio-temporal product is a result of 8-day per-pixel composition based on the product MOD11A1 (https://doi.org/10.5067/MODIS/MOD11A1.006). Images have been integrated and averaged at the monthly scale and subsequently interpolated at the level of the study area. The values are available Kelvin degree, which in turn are subsequently converted to Celsius degree.

On the other hand, the Tropical Rainfall Measuring Mission (TRMM) provides rainfall information by means of the so-called research product TRMM Multi-satellite Precipitation Analysis (TMPA) 3B42. The TMPA 3B42 was retrieved in this study at the daily time step with 0.25 degree spatial resolution. The 3B42 is based on TMPA 3B42RT (3-hourly near-real-time) with rain gauge data and is available since January 1st, 1998 [30]. More details concerning the TMPA algorithms can be found in Huffman *et al*. (2010) [31] and Huffman and Bolvin (2018) [32].

The works of consistency and formatting of the data captured by the artificial satellites were carried out by the Water Resources and Environmental Studies Laboratory (LABH2O) of the Civil Engineering Program at COPPE-UFRJ.

### The ARMAX model

In order to model the relationship between the incidence rate of dengue cases (dependent variable *y*) and climate factors (explanatory variables *x*), we chose to specify the ARMAX model, the family of autoregressive models of moving averages with exogenous variables, see for example Hamilton (1994) [33] and that in its more general form assumes the expression:

$$\begin{array}{c} y_{t}\;=\;C\;+\;\sum_{i=1}^{p}\phi_{i}\;y_{t-i}\;+\;\sum_{i=1}^{k}\beta_{i}\;X_{i,t}\;+\;\epsilon_{t}\;-\;\sum_{i=1}^{q}\theta_{i}\;\epsilon_{t-i} \end{array}$$ (1)

In Eq (1) *C* is the intercept, *ϵ*_*t*_ is an unobservable random term, *y*_*t*_ represents the value on month *t* of the monthly series of the logarithm of the incidence rate of dengue cases per 100,000 inhabitants and *x*_*t*_ represents the climate variables precipitation and temperature in the month *t*. In addition, the three coefficients *ϕ*, *θ*, *β* are not known a priori, but can be estimated from the data by means of the conditional maximum likelihood method.

From the time series of monthly dengue occurrences in the municipality of Duque de Caxias, together with the respective population estimates for July 1 of each year and the monthly rainfall and temperature series (minimum, mean and maximum), ARMAX models were separately adjusted. The specification and estimation of the coefficients of the best ARMAX models in each municipality was performed with the help of the Gretl software (http://gretl.sourceforge.net/). In the selection of the best model we tried to minimize the information criterion of Akaike. The coefficients were estimated by means of the maximum likelihood method conditioned to the observations until December 2015. The data for the year 2016 were not used in the adjustment of the models, since they formed the out-of-pocket sets used to evaluate the predictive capacity of the adjusted models based on the following metrics:

**Root of the Mean Square Error**:

$$\begin{array}{c} \text{RMSE}={\left[\frac{1}{N}\;\sum_{t=1}^{N}\;{\left(\;observed_{t}-estimated_{t}\;\right)}^{2}\right]}^{1/2} \end{array}$$ (2)

**Theil’s U**:

$$\begin{array}{c} \text{Theil}=\frac{{\left[\sum_{t=1}^{N-1}\;{\left(\;\left(\;observed_{t+1}-estimed_{t+1}\;\right)\;/observed_{t}\right)}^{2}\right]}^{1/2}}{{\left[\sum_{t=1}^{N-1}\;{\left(\;\left(\;observed_{t+1}-observed_{t}\;\right)\;/observed_{t}\right)}^{2}\right]}^{1/2}} \end{array}$$ (3)

## Results

### Temporal series of dengue cases and circulating serotypes

**Fig 1:**
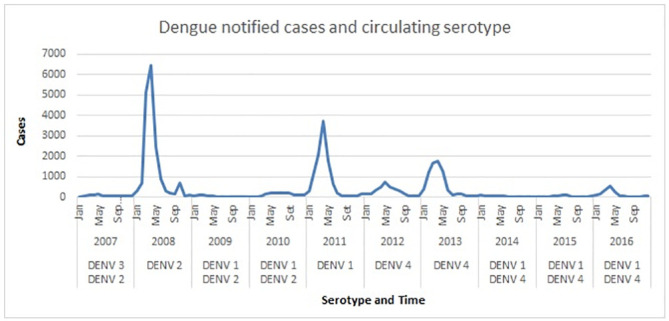
Dengue notification monthly series.

The monthly time series of reported cases of dengue in Duque de Caxias during the period from 2007 to 2016 registers a total of 43,423 cases of dengue, including epidemic and non-epidemic periods (Fig (1)). Epidemic periods were observed from January to July 2008 (16,126 cases), January to June 2011 (9,554 cases), January to August 2012 (2,855 cases) and January to June 2013 (6,689 cases). The higher number of dengue cases took place in April in the epidemic years of 2008 and 2011 and May in 2012 and 2013.

The analysis of the time series in the epidemic years shows two distinct periods. The first period is when the epidemic curve grows, coinciding with the warmest period in this region, until it reachs the apex, followed by a period of decrease of this curve. The growth rate is initially slow and, after four to six weeks, a strong increase occurs for ten to twelve weeks until it reaches the apex. Then there is a period of steep decline. Consequently, this period is always shorter, up to eight weeks. In summary, the analysis of the series of dengue cases in epidemic years shows a characteristic of seasonality, given its visible seasonal behavior, in a certain harmony with climate variables.

Analysing the information of circulating serotypes in the dengue time series, Fig 1, we observed that the DENV-3 serotype only circulated in 2007, a non-epidemic year. Thus, there is a generation of children born after that date, which is added to the adults susceptible to this serotype. Differently, the serotype DENV-1 circulated in the second major epidemic (2011) and in another five years of this time series. The DENV-2 serotype circulated in the largest epidemic (2008) and in the two following years of this time series. The DENV-4 serotype entered Duque de Caxias and caused a small epidemic in 2012 and a more intense one in 2013, and since then it still circulates. It should be noted that in the four epidemic years (namely: 2008, 2011, 2012, 2013), each year there was a dominance of only one of the four serotypes (namely: DENV-2, DENV-1, DENV-4, DENV-4).

### Time series of incidence rate logarithms

**Fig 2:**
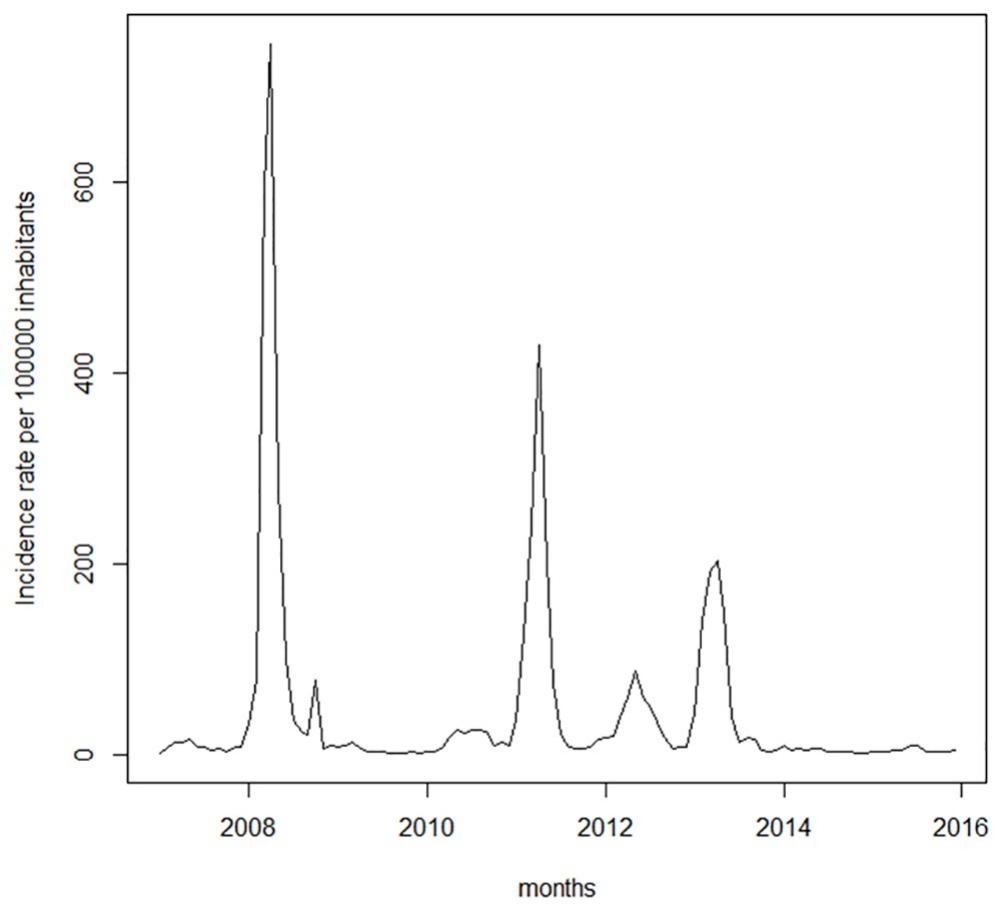
Incidence rate of dengue per 100000 inhabitants.

**Fig 3:**
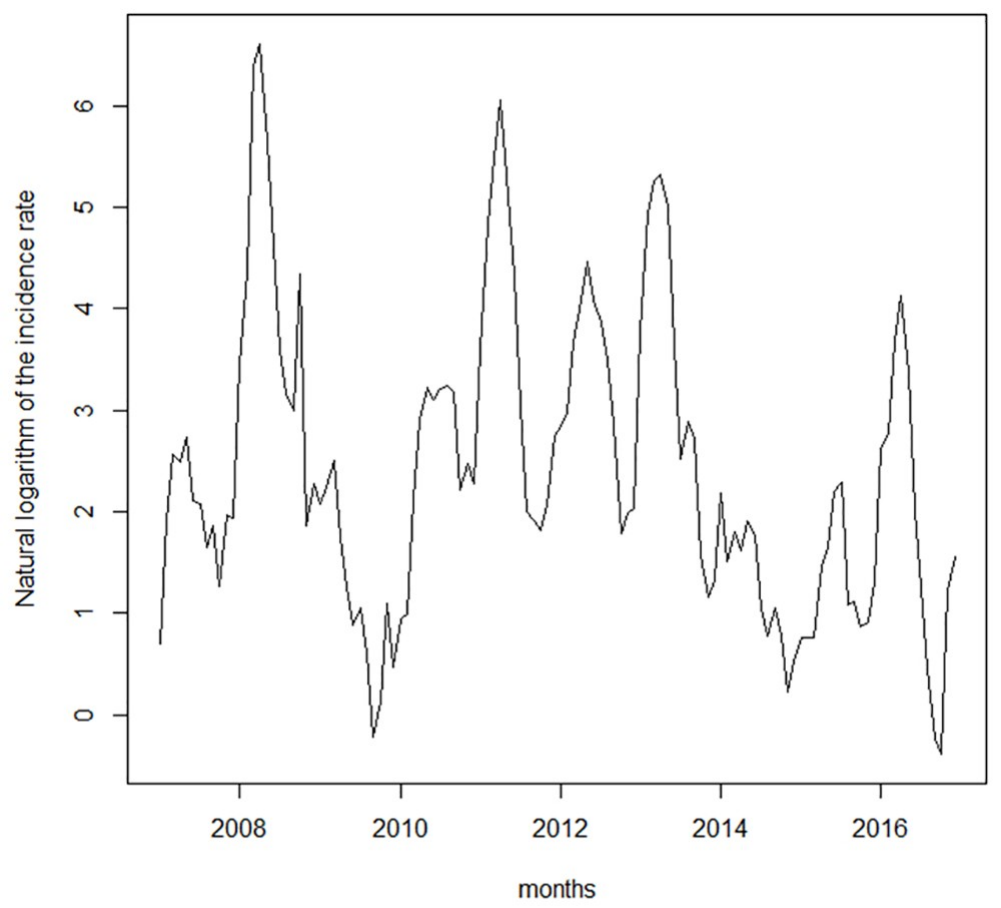
Logarithm of the incidence rate of dengue.

Fig 2 shows the time series of the incidence rate of dengue in Duque de Caxias per 100,000 inhabitants. The high peaks of occurrence suggest that the series exhibits heteroscedasticity, a characteristic that violates the premise of error homoscedasticity, which is a hypothesis assumed by the ARMAX model. For this reason, we took the natural logarithm of the incidence rate, the series of which is shown in Fig 3. The same transformation was applied by Gharbi *et al*. (2011) [21] and Guo *et al*. (2017) [22] in studies on dengue in Guadeloupe and Guangdong.

### Analysis of the influence of climate variables

In Eq (1) the time series *y* and *X* must be stationary. Therefore, based on the Augmented Dickey-Fuller Test (ADF) and KPSS test [34], a diagnosis was made to assess the stationarity of the available monthly time series, i.e., the time series of dengue incidence, temperature and rainfall. The ADF and KPSS tests indicated that the time series available are stationary. Therefore, in this case, there is no need to take time series differences. Then, after rejecting the hypothesis of non-stationarity (unit root), we started the search for the best specification of the ARMAX model capable of relating the incidence of dengue with the available climate variables. In this stage we select the explanatory variables and their respective temporal lag (lags). The identification of the best specification passes through the examination of some alternative specifications that differ in relation to the set of explanatory variables (between available variables) and their temporal lags. The best specification should meet some criteria, for example, in this work it was considered that the best specification is the one with the lowest Akaike index.

Thus, from the data from January 2007 to December 2015 (in-sample period) several specifications for the ARMAX model were evaluated. The best result specification for the ARMAX model is summarized in Table 1, from which it can be concluded that the incidence rate in one month t is explained by the rainfall (*PLUV*_1_ and *PLUV*_2_) and the logarithm of incidence rates (*y*_1_ and *y*_2_) in the immediately preceding two months (*t* − 1) and (*t* − 2).

**Table 1:** Estimated coefficients for the best ARMAX mode.

	Coeficient	Standart error	Z	p-value
*Const*	0.0433966	0.147424	0.2944	0.7685
*Phi*_1_	1.01665	0.0954977	10.65	1.82E-26
*Phi*_2_	-0.189711	0.0924959	-2.051	0.0403
*PLUV*_1_	0.00126183	0.000478276	2.638	0.0083
*PLUV*_2_	0.000917676	0.000498571	1.841	0.0657

From the coefficients estimated in Table 1, we obtain the following prediction equation:

$$\begin{array}{c} y_{t}\;=\;0.0434\;+\;1.0167\;y_{t-1}\;-\;0.1897\;y_{t-2}\;+\;0.0013\;Pluv_{t-1}\;+\;0.0009\;Pluv_{t-2}\;+\;\epsilon_{t} \end{array}$$ (4)

which can be interpreted as follows: the incidence rate *y*_*t*_ in month *t* is explained by the rates occurring in the previous two months, *y*_*t*−1_ and *y*_*t*−2_, with great predominance of the first one, as well as by the rainfall occurred during the same two months, *Pluv*_*t*−1_ and *Pluv*_*t*−2_.

**Fig 4:**
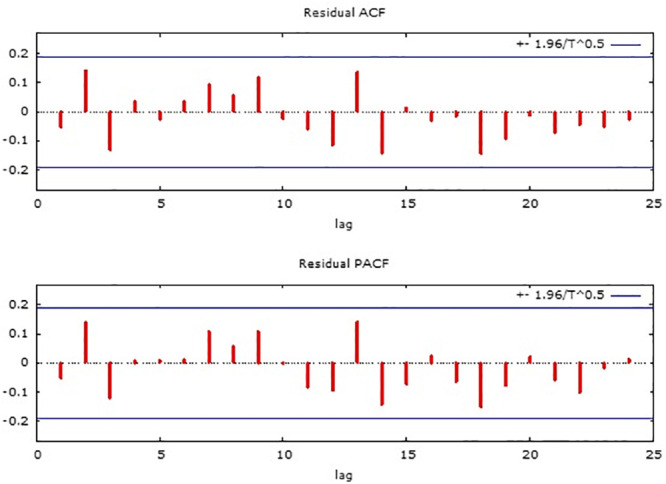
Residual ACF and residual PACF.

As illustrated by the residual ACF (Residual Autocorrelation Function) and residual PACF (Residual Partial Autocorrelation Function) in Fig 4, the residuals autocorrelations are not significant, so the model was considered satisfactory.

Table 2 shows some performance statistics of the adjusted model in the forecasted one month ahead in the out-sample period (from January to December 2016). The results for the RMSE indicators are of the same order of magnitude as other studies on the theme, such as Gharbi *et al*. (2011) [21] and Guo *et al*. (2017) [22] and the value for Theil’s U, below one, indicates that the adjusted model provides better predictions than those obtained by the naive predictor, i.e., a prediction in which the incidence rate is assumed equal to the last observed value.

**Table 2:** Performance in the out-sample period for the incidence rate.

Metric	Value
Root Mean Square Error (RMSE)	0.6744
Theil’s U	0.8597

**Fig 5:**
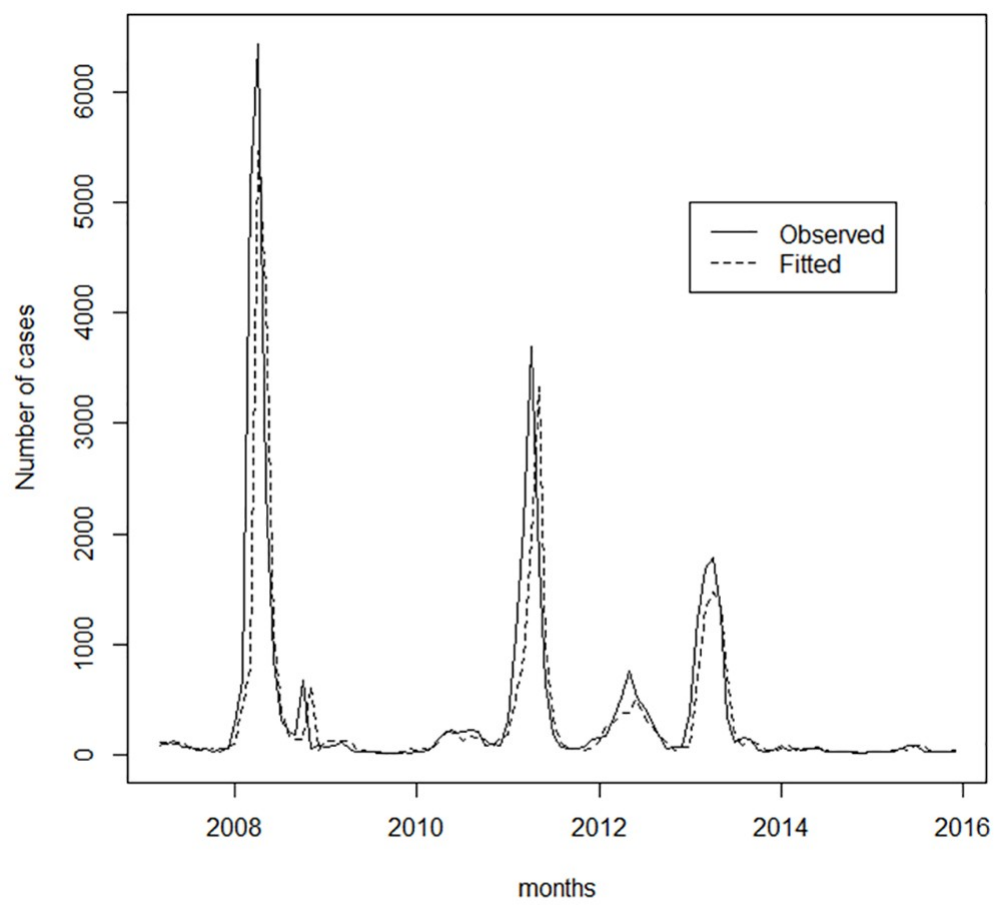
Number of cases observed versus fitted (2008-2015).

In Fig 5, the estimated cases comprise those from the beginning of the series through December 2015, where all data from that period are used in the adjustment of the equation of the ARMAX model, with which the estimated values are calculated.

**Fig 6:**
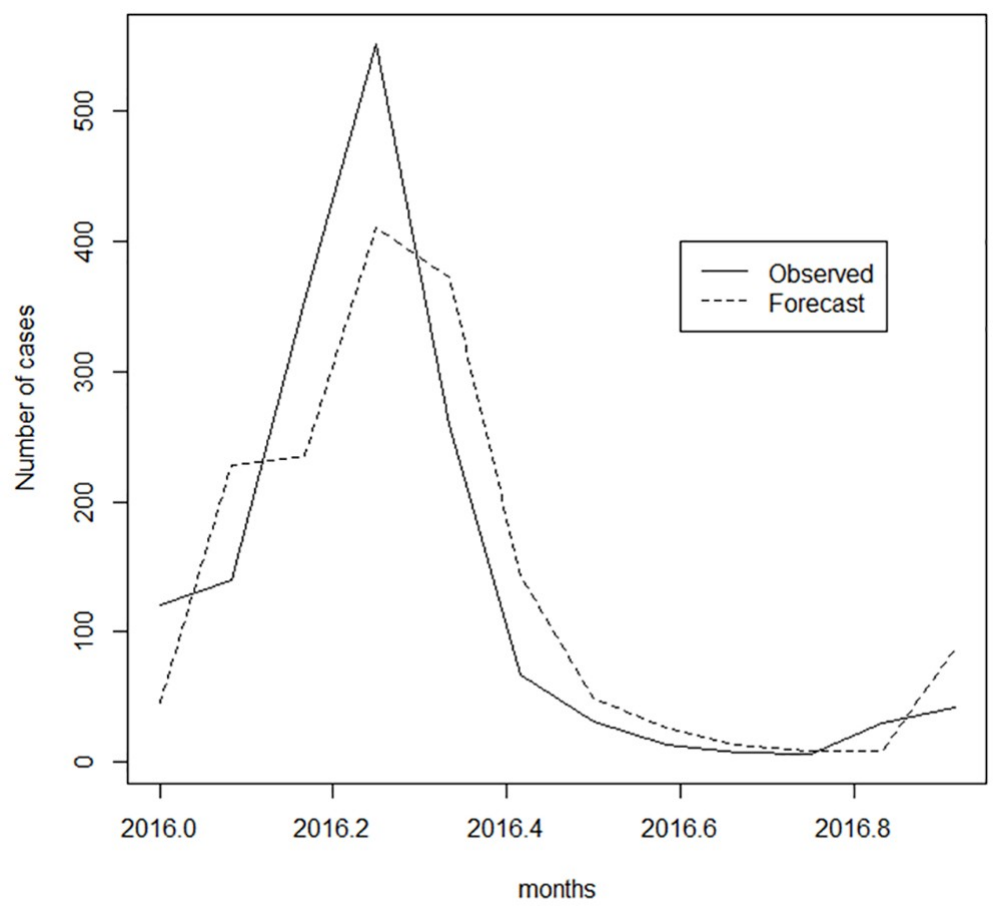
Number of cases observed versus forecast (2016).

In Fig 6, the expected cases include the out-sample period, for the months of January to December 2016, in which the ARMAX model equation is used in the calculation of forecasts one month ahead from January 2016 to December 2016. The good adherence of the model to the observed data can be visualized in Figs 5 and 6, which show the monthly observed totals of dengue occurrence and the respective fitted/forecasted values obtained from the results provided by the ARMAX model using Eq 4.

## Discussion

First, the time series of incidence rates of dengue and of rainfall are discussed separately, then a multivariate relationship between the climate variables and the incidence rates of dengue is studied. A few similar works were selected from the broad literature for a short and simplified comparison with the results shown here. All of these works show in common the importance of climate data.

Analyzing time series of the present study, it can be observed that the highest incidence rates of dengue happen in the first semester of each year. The months with the highest numbers of cases are April and May, in the Autumn season. Analyzing the rainfall chart, it can be seen that the first semester of each year is the period of greatest rainfall, the rainy season, in which the deposits of water necessary for Aedes to reproduce are created.

Gomes *et al*. (2012) studied the correlation between climate variables and the risk for dengue, analysing the period from 2001 to 2009 in the city of Rio de Janeiro. They also identified that most cases occur in the first semester, particularly between the months of March and May [17]. Their records are similar to those found in the analysis of time series of incidence rates of dengue above.

Analysing the information about circulating serotypes in the dengue time series, we observed that the DENV-3 serotype only circulated in 2007, a non-epidemic year. Thus, there is a generation of children born after that date who make up the largest contingent of people susceptible to contagion, together with the adults susceptible to this serotype. Therefore, among the four different serotypes, DENV-3 has the greater potential, in thesis, for propagation in the population and may even produce an epidemic. The diversity of serotypes of the dengue virus, the efficiency of the *Aedes aegypti* vector and their anthropophilic characteristics, coupled with the disorderly and precarious urbanization pattern of large cities in developing countries, mostly located in tropical and subtropical areas of the planet, make the control of this arbovirus infection extremely difficult and complex [13, 35].

Several studies show that breeding occurs mainly during the months of higher rainfall. Studies carried out in the Paraíba Valley (São Paulo), in São José do Rio Preto (São Paulo), in the State of Maranhão and of Paraíba, in a protected area in the metropolitan region of São Paulo, in Uberlândia (Minas Gerais) and Boa Vista (Roraima) showed that, even though there is a difference in rainfall dynamics in the various regions of Brazil, the highest incidence rates of dengue coincide with the rainy months, the hottest months of the year in the country [36–44]. The density and availability of breeding sites for Ae. Aegypti, determined by the regime of rainy and dry periods, exert great influence in the dispersion of this species [3].

Honorio *et al*. (2009) offer explanations for the conclusions of previous studies. They report that the occurrence of weekly average temperatures above 22°–24°C is strongly associated with high *Aedes aegypti* population and consequently with an increased risk of dengue transmission [45]. The rainfall factor is important for the production of larvae, pupae and mosquitoes, influencing the occurrence of dengue. The variability of precipitation interferes in the breeding of larvae and pupae of *Aedes aegypti*. Rainfall interacting with temperature affects evaporation, thus affecting the breeding water viability, see also Morin *et al*. (2013) [46].

Viana and Ignotti (2013) systematically review 31 articles published in databases (Scielo, PubMed and Lilacs) concerning dengue and meteorological variations in Brazil between 1991 and 2010. In a summary of the 31 papers, the authors record that in ten of these articles there is a correlation between the occurrence of dengue only with the variable rainfall, whereas in only four articles, there is correlation only with the temperature variable [13].

Struchiner *et al*. (2015) specified a multivariate Poisson regression model to explain the incidence of dengue fever in Singapore. Population growth, Ae aegypti population, climate parameters such as temperature and rainfall, and population arrivals of endemic dengue regions are defined as variables for the model. This model estimates the values of the contribution of these individual factors in increasing the incidence of dengue. The model concludes that the predominant individual factor for the occurrence of dengue was population growth, followed by climate factors [47].

Sharmin *et al*. (2015) in modelling the dengue occurrence series, in the Dhaka metropolitan region, Bangladesh, apply a generalized (binomial negative) linear regression model. The authors analyse the relationship between dengue and climate. To do so, they include temperature and rainfall as explanatory variables in the prediction equation of dengue incidence [48].

Chuang *et al*. (2017) model the dengue time series in Taiwan using distributed lag non-linear regression models. The authors evaluated several variable specifications for the prediction equation [25]. The best model found has as explanatory variables: minimum temperature and precipitation.

Koh *et al*. (2018) adjusted a neural network and a Poisson regression model to weekly data on dengue incidence and precipitation in Singapore. In the models presented by the authors, the incidence of dengue is explained according to the past values of the incidence rate and precipitation [49].

The present study *a priori* is also based on the temperature and precipitation climate variables, ubiquitous in the set of works listed above. However, the importance of the remaining variables cannot be ruled out, such as, for example, the already commented expressive flow of people in Duque de Caxias, which was not verified due to the absolute absence of data.

The statistical model used in this work for joint analysis of the incidence series and climate variables was the ARMAX (Auto-regression Integrated Moving Average Cause Effect), which is a hybrid model, since it has characteristics of strictly temporal model conjugated with multiple regression, to contemplate other explanatory variables.

In an initial exploratory phase, the low explanatory power of the temperature variable was identified. After a few attempts of using only the effect of precipitation, the final formulation definition was obtained, having as explanatory variables the rainfall and the incidence rates of dengue in the two previous months.

As shown in Fig 1, the highest incidences of dengue occurs up to 2 months after the end of summer, a result consistent with the lags (*t* − 1) and (*t* − 2) of rainfall, identified in the final formulation of the ARMAX model.

The final formulation reproduced the incidence series of dengue with very good accuracy. As a more remarkable result, this same formulation was also able to produce forecasts a month ahead in the out-sample period (from January to December 2016), which were very close to the values actually observed in the step ahead.

In order to show the strength of the approach developed in the present study, some of its properties and results should be highlighted.

The first property is simplicity, since the model is simple, natural, orthodox, because it is based on climate variables, and very accessible to a good range of health managers, even in small municipalities. Likewise, the mathematical formulation employs one of the most widespread techniques in statistics and multivariate analysis, available through several providers of free software through the internet, which facilitates its use.

Since this work was based on climate data coming from remote sensing via artificial satellites, previously available cost-free for the whole world, without the deleterious feature of having missing values, this approach offers a great range of applicability, in particular for poor countries located in tropical regions where dengue is widespread.

Regarding adhesion to field data, the developed model was able to overcome difficulties common to other alternatives, producing predictions with an adequate precision. As to its predictive capacity, it should be highlighted that the developed model was able to accurately predict the incidence rate with a horizon of one month ahead during a period of twelve months.

Regarding the possibility of generalization, the developed model can also be applied to other arbovirus diseases, in particular chikungunya and zika, which have *Aedes* as a vector.

Taking into account that there are historical deficiencies in the mechanisms of arbovirus surveillance, this work could be replicated in several cities around the world by public health managers, to build auxiliary operational tools for the tasks of control and prevention.

The ARMAX model was adjusted from the dengue incidence rates, rainfall and temperature. The most critical limitation of the study is the monthly periodicity of series. In epidemic periods, the variation of incidence rates can be large, which can produce extremely high incidence rates values, as shown by Fig 1 in the year of 2008. Therefore, thirty days is a very long time interval, one in which the vector population can explode. Thus, it is advisable to adopt a shorter time interval, as a weekly or daily one, to allow more efficient and fast prevention actions by the public health managers. Moreover, the inclusion of other explanatory variables can improve the performance the ARMAX model.

## Supporting information

S1 Data This file contains data shown in Fig 1. (XLSX)

S2 Data This file contains data shown in Fig 2. (XLSX)

S3 Data This file contains data shown in Fig 3. (XLSX)

S4 Data This file contains data shown in Fig 5, as well, it is used for calculation of the values, residual ACF and Residual PACF, shown in Fig 4. (XLSX)

S5 Data This file contains data shown in Fig 6. (XLSX)

S6 Data This file contains the complete set of data shown in figures and, as well, it is used by ARMAX model, where variables *y*_*t*_ and *Pluv*_*t*_ correspond respectively to columns C and D of this file. (XLSX)
